# Feasibility of Using Gluconolactone, Trehalose and Hydroxy-Propyl Gamma Cyclodextrin to Enhance Bendroflumethiazide Dissolution Using Lyophilisation and Physical Mixing Techniques

**DOI:** 10.3390/pharmaceutics10010022

**Published:** 2018-02-01

**Authors:** Ashraf Saleh, Kenneth McGarry, Cheng Shu Chaw, Amal Ali Elkordy

**Affiliations:** School of Pharmacy and Pharmaceutical Sciences, University of Sunderland, Sunderland SR1 3SD, UK; bg44ce@research.sunderland.ac.uk (A.S.); ken.mcgarry@sunderland.ac.uk (K.M.); cheng.chaw@sunderland.ac.uk (C.S.C.)

**Keywords:** bendroflumethiazide, hydrophobic drug, lyophilisation, gluconolactone, hydroxyl propyl γ-cyclodextrin, trehalose, dissolution rate

## Abstract

Purpose: Hydrophobic drugs are facing a major challenge in dissolution rate enhancement and solubility in aqueous solutions; therefore, a variety of methods have been used to improve dissolution rate and/or solubility of bendroflumethiazide as a model hydrophobic drug. Methods: In this study, two main methods (physical mixing and lyophilisation) were used with gluconolactone, hydroxyl propyl γ-ccyclodextrin, and trehalose to explore this challenge. Bendroflumethiazide, practically insoluble in water, was mixed with one of the three excipients gluconolactone, hydroxyl propyl γ-cyclodextrin, and trehalose in three different ratios 1:1, 1:2, 1:5. To the best of our knowledge, the dissolution of the drug has not been previously enhanced by using either these methods or any of the used excipients. Samples containing drug and each of the excipients were characterized via dissolution testing, Fourier Transform infra-red spectroscopy, differential scanning calorimetry, and scanning electron microscopy. Results: The used methods showed a significant enhancement in dug dissolution rate; physical mixing significantly, *p* < 0.05, increased the percentage of the drug released with time; for example, bendroflumethiazide dissolution in distilled water was improved from less than 20% to 99.79% within 90 min for physically mixed drug-cyclodextrin 1:5. The lyophilisation process was enhanced and the drug dissolution rate and the highest drug dissolution was achieved for (drug-gluconolactone 1:1) with 98.98% drug release within 90 min. Conclusions: the physical mixing and freeze drying processes significantly increased the percentage of drug release with time.

## 1. Introduction

For the pharmaceutical industry, one of the greatest challenges in the hydrophobic drug development process is the water solubility, which is the key controlling dissolution rate, and hence bioavailability [1]. Drug must be dissolved in the gastric fluids to be absorbed into the systemic circulation following oral administration, incomplete absorption from the gastrointestinal tract leads to disruption in drug distribution, reduced bioavailability and therapeutic failure [2]; hence, solubility enhancement for hydrophobic drugs is essential. Oral intake is the most convenient and common route of drug delivery because of its ease of administration, high patient compliance, cost effectiveness, least sterility restraint, and flexibility in the design of dosage forms. Therefore, a lot of drug companies are obsessed with producing bioequivalent oral drug products. However, the major challenge with the design of oral dosage forms lies with their poor dissolution and bioavailability. The oral bioavailability relies on a few factors including aqueous solubility, drug permeability, dissolution rate, and metabolism. The most frequent causes of low oral bioavailability are accountable to poor solubility and low permeability.

Solubility is an important element to achieve the desired concentration of drugs in the systemic circulation for achieving required pharmacological response. Poorly water soluble drugs often require high doses to reach therapeutic plasma concentrations after oral administration. Low aqueous solubility is the major problem encountered with formulation development of new drugs and generic development [3]. To overcome this problem there are many techniques for: (i) solubility and/or dissolution enhancement including, primarily: particle size reduction (see for example [4]), co-solvents [5], pH adjustment [6], and (ii) dissolution rate enhancement including mostly: liquisolid techniques (e.g., in [7,8,9,10]), solid dispersions [11], freeze drying [12], spray drying [13], emulsions [14], nanosuspentions [15], liposomes [16], surfactant systems [17], complexation with cyclodextrins [18], and hot-melt extrusion [19].

The choice of the enhancement method is crucial and depends on the drug and excipient physicochemical properties. Lyophilisation has been used to enhance the solubility of some hydrophobic drugs such as nifedipine [20]. Freeze drying can be used for formulations development of heat sensitive drugs, where other heat base methods like hot-melt extrusion might fail [21].

Bendroflumethiazide is the active ingredient used in this research, the drug is practically insoluble in water, soluble in ethanol; it is a diuretic, antihypertensive agent for blood pressure treatment, it is available in the market as 2.5 mg and 5 mg dose tablets [22]. There were attempts in the past to enhance this drug solubility/dissolution including spray drying process [12,23], using solid dispersion formulations with polymers such as polyvinylpyrrolidone, polyethylene glycols [24]; however, these polymers have certain limitations, such as a tendency to crystallise and an inability to stabilise some active ingredients in the solid phase [25]. In this current study, bendroflumethiazide with various sugar molecules-based dissolution enhancing carriers (namely, hydroxyl-propyl gamma cyclodextrin, gluconolactone and trehalose) were processed by the lyophilisation and physical mixing techniques. Sugars are known to enhance dissolution of poorly water-soluble drugs [26], however, this is based on drugs’ chemical structures hence each drug is unique in its own. To the best of our knowledge, based on a literature search, there has been no attempt to enhance bendroflumethiazide dissolution using physical mixing and freeze drying methods with the chosen sugar molecules-based excipients. 

Cyclodextrin: (2-hydroxypropyl)-γ-cyclodextrin is a family of cyclic oligosaccharides with a hydrophilic outer surface and a lipophilic central cavity, with toroidal or cone shaped, as a result of their molecular structure and shape, they possess a unique ability to act as molecular containers by entrapping guest molecules in their internal cavity. Typical cyclodextrins, contain a number of glucose monomers ranging from six to eight units in a ring molecular, they are α (alpha)-cyclodextrin: 6-membered sugar ring, β (beta)-cyclodextrin: 7-membered sugar ring, γ (gamma)-cyclodextrin: 8-membered sugar ring [27], cyclodextrin has been used successfully as a solubiliser for many hydrophobic drugs such as furosemide, diclofenac, ketoconazole [28].

Gluconolactone is a polyhydroxy acid, lactone or oxidized derivative of glucose, a white odourless crystalline powder, freely soluble in water. It was utilized as a potential carrier to improve the dissolution rate of carbamazepine from physical mixtures and solid dispersion formulations [29]. 

Trehalose is a disaccharide formed by a 1,1-glucoside bond between two α-glucose units, freely soluble in water, and used as a carrier to increase protein stability, trehalose is used with protein formulations to prevent degradation and to enhance proteins’ stability [30] and it was used in this study to investigate its effect on drug dissolution.

The aim of this study was to evaluate the effectiveness of the above-mentioned excipients with physical mixing and lyophilisation techniques on dissolution performance of bendroflumethiazide, by tracking morphological and physicochemical drug characteristics during this enhancement process. 

## 2. Materials and Methods

### 2.1. Materials

Bendroflumethiazide is a white crystalline powder, obtained from Sigma-Aldrich (Dorset, UK). Cyclodextrin: (2-hydroxypropyl)-γ-cyclodextrin, gluconolactone is a polyhydroxy acid (PHA), and trehalose (a disaccharide a white odourless crystalline powder) were obtained from Sigma-Aldrich (Dorset, UK). All materials and chemicals were of analytical grade and used as obtained.

### 2.2. Methods

#### 2.2.1. Calibration Curve

**Figure 1:**
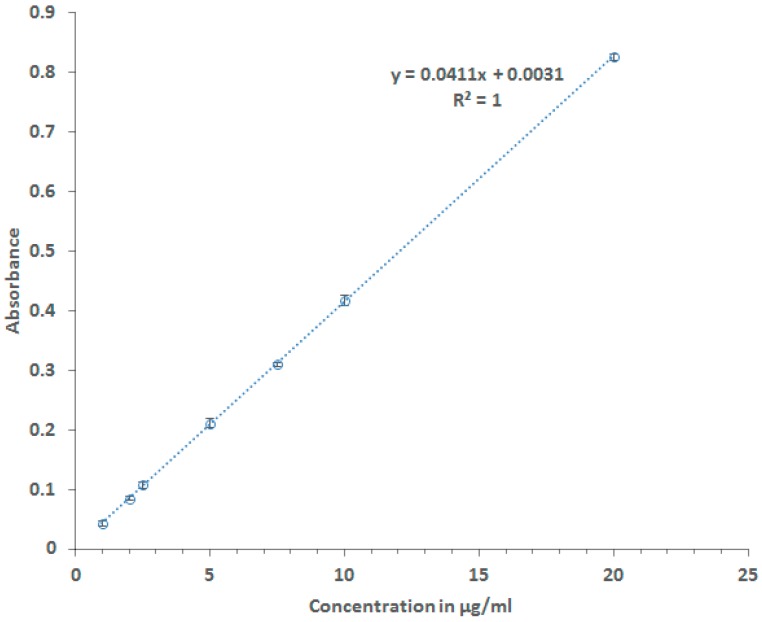
Bendrofluromethiazide Calibration Curve.

The calibration curve is needed to calculate the concentration of the drug. The calibration curve (Figure 1) was constructed (by dissolving the drug in a solution of 0.1 M NaOH) to establish the equation needed to calculate the drug concentration in relative to its absorbance at wavelength 273 nm using M501 single beam scanning ultra-violet spectrophotometer, Spectronic Tudor (Leeds, UK).

#### 2.2.2. Physical Mixing

In physical mixing, the drug, bendroflumethiazide, was mixed with one of the three chosen excipients, (2-hydroxy-propyl)-γ-cyclodextrin, gluconolactone, trehalose, in three different drug-excipient *w*/*w* ratios: 1:1, 1:2, 1:5 using mortar and pestle. A pestle and mortar were used in the mixing process and a standard operation procedure similar to that of extemporaneous dispensing powders’ manufacture was followed. If sequential building up the amount of ingredients in the mortar took place, then the drug was initially mixed with an approximately equal amount of diluent (excipient), and a further amount of the diluent, equal to the amount of materials in the mortar, was added and mixed. This process was continued until the full amount of the excipients had been added, then the full contents were mixed in the mortar for 10 min. This process resulted in a form of neutral mixture (the constituents have no tendency to mix freely or segregate spontaneously once work has been input to mix them [31]) or mixture where two or more substances are physically mixed but not chemically combined. This means that the change can be reversed.

#### 2.2.3. Lyophilisation-Freeze Drying

Lyophilisation or freeze-drying process, is a dehydration process used to preserve a perishable material. Freeze-drying works by freezing the material and then reducing the surrounding pressure to allow the frozen water in the material to sublimate directly from the solid phase to the gas phase. Once the ice is sublimated, the contents of the solutions are freeze-dried and can be removed from the freeze dryer. In this research, lyophilisation was conducted on powdered formulations, by dissolving the powder admixtures in ethanol: water (50:50, this ratio was chosen as it resulted in complete solubility of the powders), and then the solutions were subjected to freezing at −85 °C, then lyophilised using a freeze drier (VirTis freeze drier, BioPharma, Boston, MA, USA).

## 3. Characterisation of Physically Mixed and Lyophilised Bendroflumethiazide Samples

### 3.1. Dissolution Studies

In vitro drug dissolution studies were performed on pure drug (5 mg) and each of the lyophilised and physically mixed formulations equivalent to 5 mg drug in hard capsules size 0, using USP dissolution apparatus II (Caleva Ltd., Dorset, UK). In this method, distilled water was used as a dissolution medium. The capsules containing formulations were placed in a dissolution medium of 900 mL at a temperature of 37 ± 1 °C and stirred at a paddle speed of 100 rpm. Moreover, ten millilitres of the samples were collected at intervals 5, 10, 15, 20, 25, 30, 45, 60 and 90 min and were replaced by 10 mL (an equal amount) of distilled water to maintain a constant volume. Then the collected samples were analysed via UV spectrophotometer at 273 nm for determination of the drug content using the calibration curve (Figure 1); the absorbance of the used excipients in the dissolution medium at 273 nm has been checked and there was no absorbance interference from those excipients. The dissolution experiment was performed in triplicate to compare the percentage drug released from lyophilised or physically mixed formulations and pure drug forms.

### 3.2. Fourier Transform Infra-Red Spectroscopy (FT-IR)

After freeze-drying or physical mixing, where the drug and three different vehicles were mixed in different ratios, it was essential to confirm how the structure and finger print area of the drug was affected during these processes. Fourier Transform Infra-Red spectroscopy (FT-IR) was performed using SHIMADZU (Buckinghamshire, UK), class 1 laser product (Buckinghamshire, UK). The spectra were measured over the range between 4000–500 cm^−1^, and the resolution used was 4 cm^−1^. FT-IR has two main advantages: firstly, the results are obtained very quickly, and secondly, the required amount of the sample is relatively small.

### 3.3. Scanning Electron Microscopy—SEM

SEM, Hitachi S3000N model (Hitachi High-Technologies UK-Electron Microscopes, Wokingham, Berkshire, UK) was used to investigate the effect of the freeze drying process on the surface morphology of the powdered formulations. Alongside other analyses, a comparison of the shape and morphology of pure drug crystals, before and after lyophilisation, helps to make further interpretations and gain a deeper understanding the drug dissolution rate enhancement after freeze drying process. Each sample was attached, using a double sided carbon adhesive tab, to a 15 mm diameter aluminium specimen stub (Mikrostik adhesive, Agar Scientific, Stansted, UK), then the samples were sputter-coated with a mixture of thin layer Gold/Palladium to allow samples to be electrically conductive. This was carried out using a Quorum technology (Polaron range) SC760 (East Sussex, UK), whereby the samples are exposed to Argon atmosphere at 10 Pa. The samples were coated at a process current of 18–20 mA for 2 × 105 s with a turning through 180 degrees in between, the magnification was ×200.

### 3.4. Differential Scanning Calorimetry—DSC

Differential scanning calorimetry, or DSC, is a thermal analysis technique in which the difference in the amount of heat required to increase the temperature of a sample and reference is measured as a function of temperature. Both the sample and reference are maintained at nearly the same temperature throughout the experiment. Generally, the temperature programme for a DSC analysis is designed such that the sample holder temperature increases linearly as a function of time. The reference sample should have a well-defined heat capacity over the range of temperatures to be scanned. The used DSC was Q 1000 TA (TA Instruments, Ghent, Belgium). The DSC was calibrated for temperature using pure Indium standard. DSC hermetically sealed aluminium sample pans were used with powdered formulations weighing about 3 mg and loaded under nitrogen at a flow rate of 50 mL/min. The pans were scanned from 0 °C–300 °C, with a rate of 10.0 °C/min. 

### 3.5. Statistical Analysis

The *t*-test was applied and results are quoted as statistically significant when *p* < 0.05.

## 4. Results and Discussion

### 4.1. Dissolution Studies

**Figure 2:**
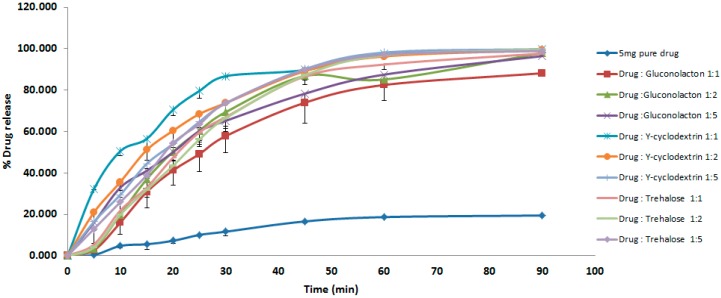
Dissolution studies from Physical mixing.

For physically mixed samples, the dissolution studies (Figure 2) show that physical mixing significantly increased the percentage of drug release with time, where the *p* value was <0.05, with nearly a 100% release within an hour, compared with just less than 20% for the pure drug without enhancing excipients. All drug vehicles formulas showed more than 55% release within 30 min and by the end of this test, after 90 min the dissolution extents were as follows: (drug-cyclodextrin 1:5 (99.79%)), (drug: trehalose 1:2 (99.69%)), (drug: gluconolactone 1:2 (97.85%)). Gluconolactone has been used to be a potential hydrophilic carrier to improve the dissolution rate of a hydrophobic drug such as carbamazepine, using physical mixtures and solid dispersion formulations [29]. Moreover, gluconolactone improved the dissolution rate of a poorly water soluble piroxicam [32]. Cyclodextrins were used as solubilisers and successfully enhanced poorly soluble drugs (as a result of complex formations due to their unique molecular structures which act as containers for entrapping guest molecules in their internal hydrophilic cavities) such as Norfloxacin, Meloxicam, Celecoxib, Valsartan, Glibenclamide and many others [17,28,33], To the best of our knowledge, Trehalose has not been used as a drug solublizer before, and its dissolution enhancements for bendroflumethiazide show a promising opportunities to be used with other poorly soluble drugs. Generally, physical mixing of the drug and excipients did not exhibit significant dissolution improvement; however, in this study, the chosen excipients with the physical mixing process were successful at enhancing bedroflumethazide dissolution.

**Figure 3:**
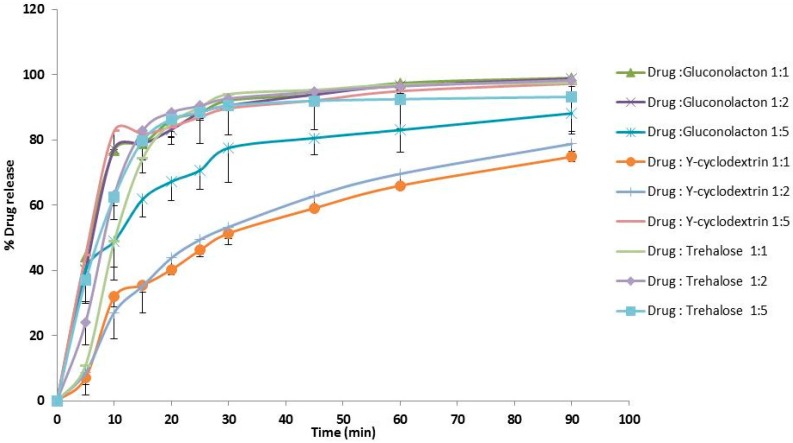
Dissolution studies after Lyophilisation-Freeze drying.

For freeze dried drug samples, there has been a considerable improvement for drug dissolution rate (Figure 3) via lyophilisation process within quiet short period; 66–97% of the drug was released within an hour, compared to 20% for the pure unprocessed drug. Gluconolactone and trehalose were superior over cylodextrin at its lower ratios. After 90 min the dissolution profiles reached 99.75% for (drug-cyclodextrin 1:5); 98.98% for (drug-gluconolactone 1:1); 97.23% for (drug-cyclodextrin 1:5) and 93.18% for (drug-trehalose 1:5). There were no significant differences (*p* > 0.05) for drug dissolution profiles using gluconolactone at all used ratios 1:1, 1:2 and 1:5. Also, there were no significant differences (*p* > 0.05) for drug dissolution profiles using trehalose at all used ratios. However, there were significant differences (*p* < 0.05) for drug dissolution using drug-cyclodextrin 1:1 or 1:2 versus drug-cyclodextrin 1:5. 

### 4.2. Fourier Transform Infra-Red Spectroscopy (FT-IR) Results

**Figure 4:**
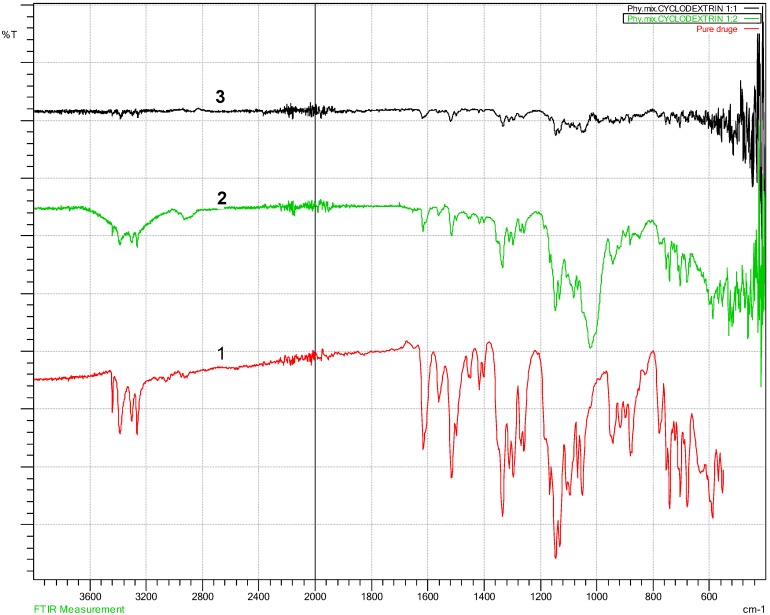
Drug-cyclodextrin Fourier Transform Infra-Red spectroscopy (FT-IR) showing no change on peak locations, but there is a significant change on peak intensities. 1 (red colour): Pure bendroflumethiazide; 2 (green colour): Drug: cyclodextrin 1:2; 3 (black colour): Drug: cyclodextrin 1:1.

**Figure 5:**
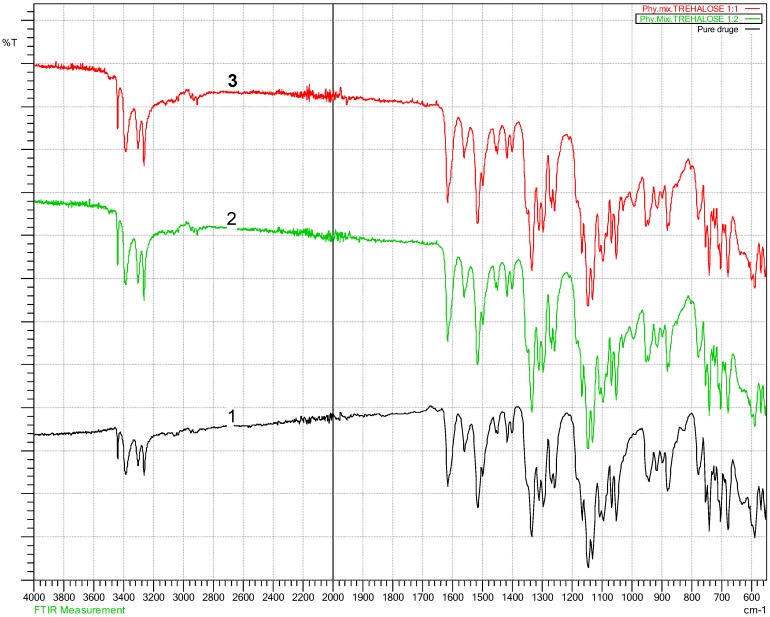
Drug-trehalose FT-IR showing no change on peak locations, but there is a significant change on peak intensities. 1 (black colour): Pure bendroflumethiazide; 2 (green colour): Drug: trehalose 1:2; 3 (red colour): Drug: trehalose 1:1.

**Figure 6:**
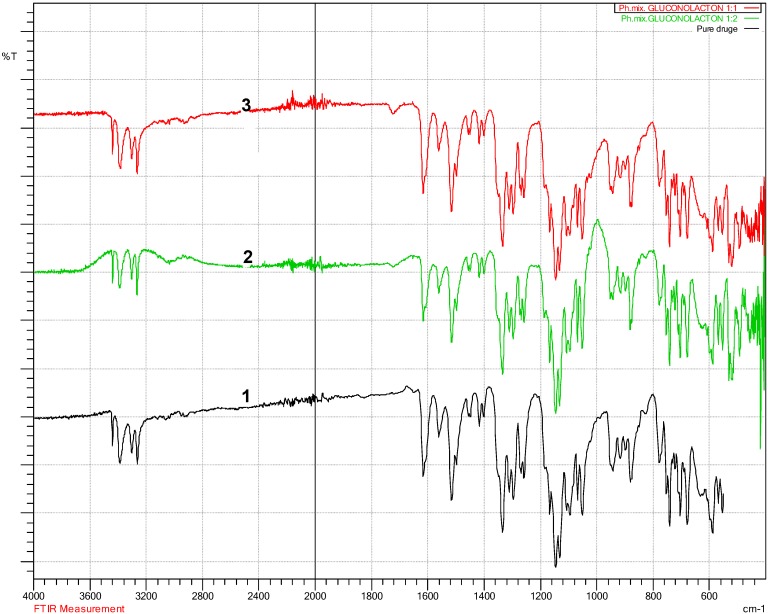
Drug-gluconolactone a new peak (marked with a circle) appeared between 1715–1730 cm^−1^ due to ketone functional group C=O stretching. 1 (black colour): Pure bendroflumethiazide; 2 (green colour): Drug: gluconolactone 1:2; 3 (red colour): Drug: gluconolactone 1:1.

The FT-IR is essential to establish any changes within characteristic functional group area, peaks movement, reduction, shifting or any altering to the characteristic peaks within finger printing area (a specific unique characteristic region of peaks for each compound in the FT-IR spectrum) should be notified. These changes reflect what happened to specific functional group, and how they were affected to that change. According to Beer’s law, the absorptivity of a material is related to the concentration of that material. Concentration is very important for the peak intensity; bonds between atoms vibrate in a specific way depending on other connecting atoms and their concentration [34]. The FT-IR test for physically mixed and lyophilised samples (Figure 4, Figure 5 and Figure 6) did not show any shifting in the peaks location (Figure 4 and Figure 5), a part from drug-gluconolactone physical mixtures and lyophilised samples. A new peak appeared between 1715–1730 cm^−1^ due to ketone functional group C=O stretching (Figure 6), but the intensity varied depending on the added excipients and their ratios. The FT-IR spectrum of the drug showed reduced peak intensity after adding gluconolactone, cyclodextrin, and trehalose in ratio 1:1, 1:2, 1:5. The drug’s peak intensities reacted in inverse proportion to the concentration of the excipients, and proportionally to a mount of the drug, i.e., for higher drug-excipient ratios, the amount of drug was less, therefore, the drug peak intensities were reduced. A similar phenomenon occurred in the literature, supporting the explanation that, when the concentration of carriers in solid dispersion formulations of carbamazepine-gluconolactone increased to 1:4, the intensity of the drug peak significantly reduced, and it was similarly explained that this could be due to the presence of less carbamazepine in these samples [29]. Dissolution studies supported this phenomenon as the dissolution rate was higher when the excipients ratio was increased. The spectra of drug-cyclodextrin and drug-gluconolactone, show how significantly the peak intensity was reduced compared to the spectrum of pure drug, meaning that excipients may solubilise the drug within them at the molecular level to large extent as confirmed by DSC data as well. Drug-cyclodextrin peaks in the finger print area between 3263.56 cm^−1^–3439.08 cm^−1^, and characteristically for primary Amine (N–H) stretching, the vibration was reduced significantly in direct proportion to the concentration. In the range between 1516.05 cm^−1^–1616.35 cm^−1^ there were significant reductions for the aromatics in ring C–C stretching and other clear reductions in the area between 1250 cm^−1^–1335 cm^−1^ C–N aromatic amine stretching [34].

### 4.3. Differential Scanning Calorimetry (DSC)

**Figure 7:**
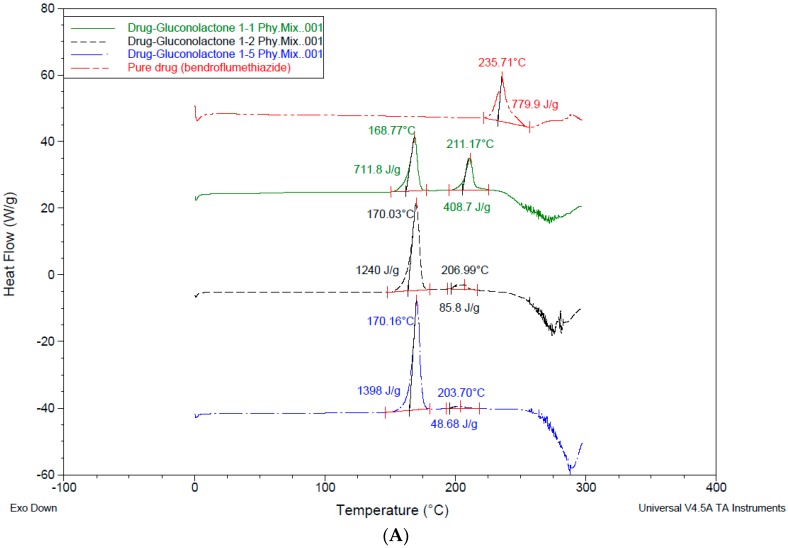

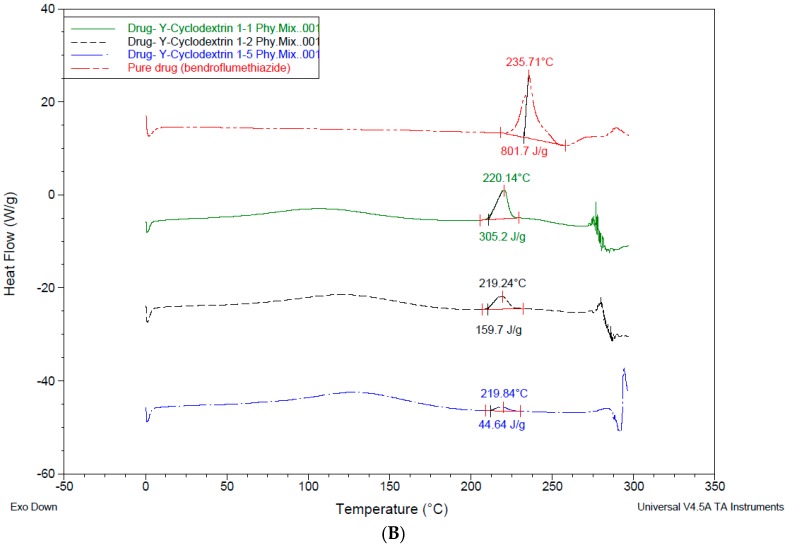

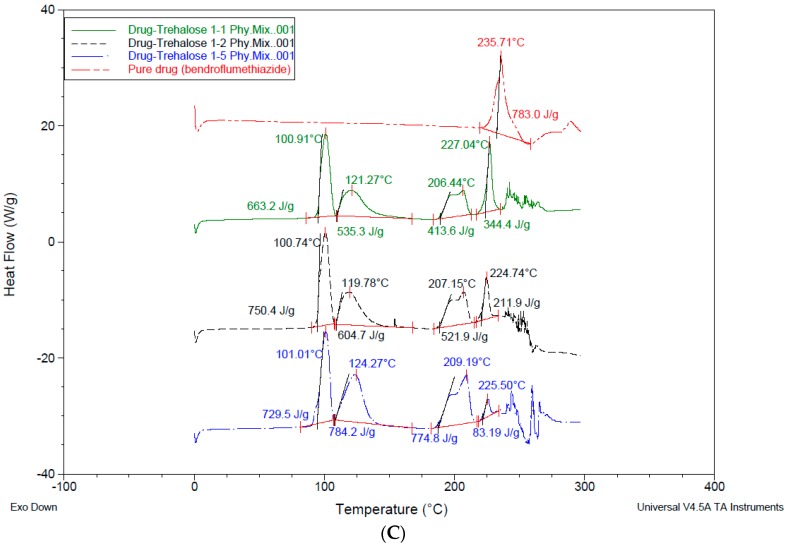
Differential Scanning Calorimetry (DSC) data for physically mixed drug with excipients. (**A**) DSC for drug-gluconolactone after physical mixing; (**B**) DSC for drug-hydroxyl propyl γ-cyclodextrin after physical mixing; (**C**) DSC for drug-trehalose after physical mixing.

DSC was used for the investigation of any interaction between the drug and the used excipients [6,20,35]. The DSC thermogram for the pure drug shows a sharp endothermic peak at 235.71 °C corresponding to its melting point *T*m, and a high enthalpy of 769.81 J/g. This peak is an important indication of any changes to the physical or chemical characteristics of peak loss or peak shifting; this indicates the drug’s change in nature. The Bendroflumethiazide peak was shifted to lower *T*ms from 235.71 °C as a pure drug to 211.17 °C, 206.99 °C, 203.70 °C after physical mixing with gluconolactone 1:1 1:2, 1:5 respectively and a reduction to the enthalpy was shown (Figure 7A). The significant reduction in *T*m and enthalpy energy after physical mixing does indicate that the drug crystallinity was reduced with the presence of excipients or some of the drug was soluble at a molecular level in the excipient. In addition, a eutectic mixture of bendroflumethiazide and gluconolactone may be formed [29], and from the literature, eutectics have been shown to have many advantages including improved thermodynamic functions due to greater molecular mobility, high free energy, and weaker intermolecular interactions. Therefore, they should exhibit high solubility and faster dissolution [36,37]. Dissolution testing supports this result, as gluconolactone shows a significant improvement in the dissolution of the drug. The drug behaviour with cyclodextrin physical mixing was not different (Figure 7B), *T*m shifted to lower temperatures and the enthalpy reduction was up to 305.25 J/g, 159.7 J/g, 44.64, J/g in drug -excipient ratio 1:1, 1:2, 1:5 respectively (Figure 7B), suggesting a reduction in the drug crystallinity and hence improvement of its dissolution profile. In the same way, DSC thermograms for trehalose samples have approved the enhancement for the drug dissolution, as *T*m and enthalpy were reduced (please refer to Figure 7C).

**Figure 8:**
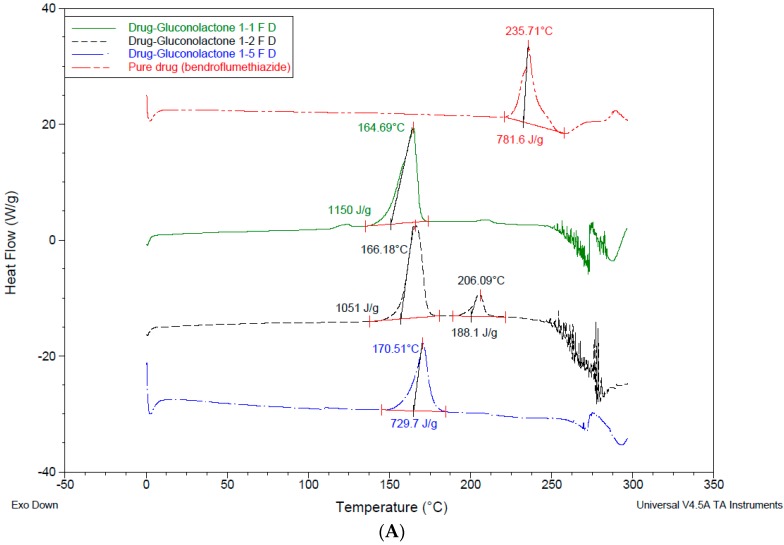

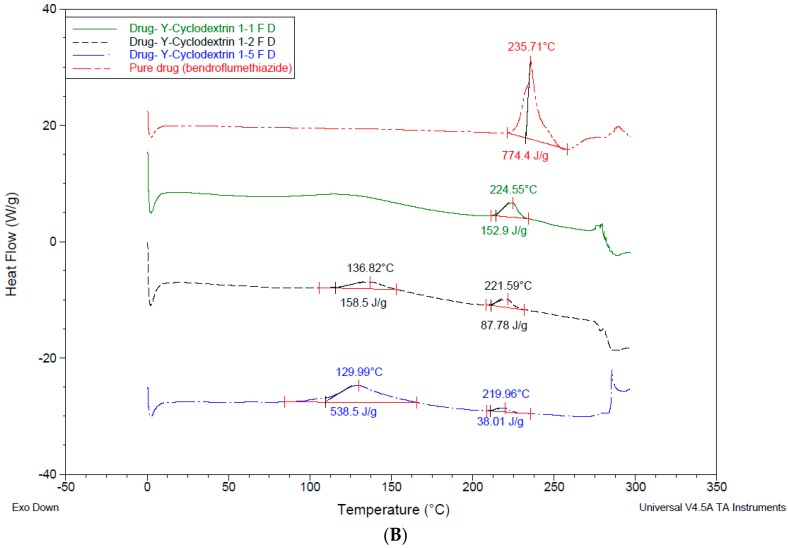

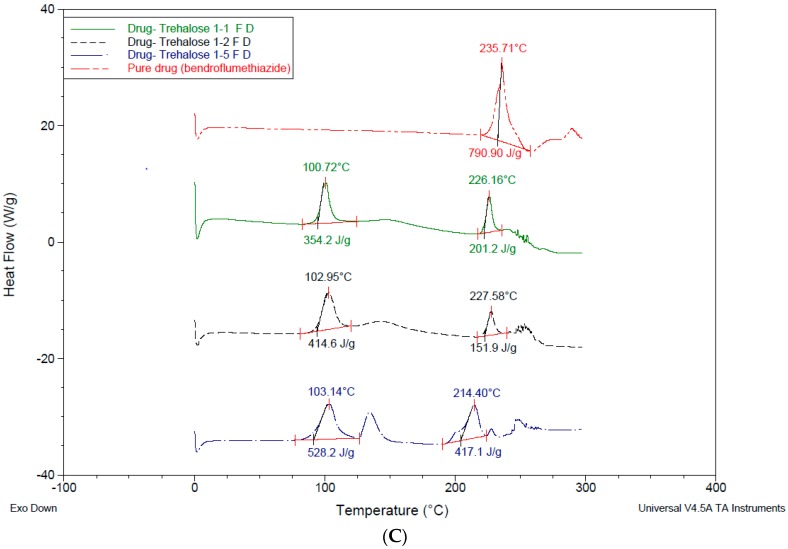
DSC data for lyophilised drug with excipients. (**A**) DSC for drug-gluconolactone after freeze drying; (**B**) DSC for drug-hydroxyl propyl γ-cyclodextrin after freeze drying; (**C**) DSC for drug-trehalose after freeze drying.

**Figure 9:**
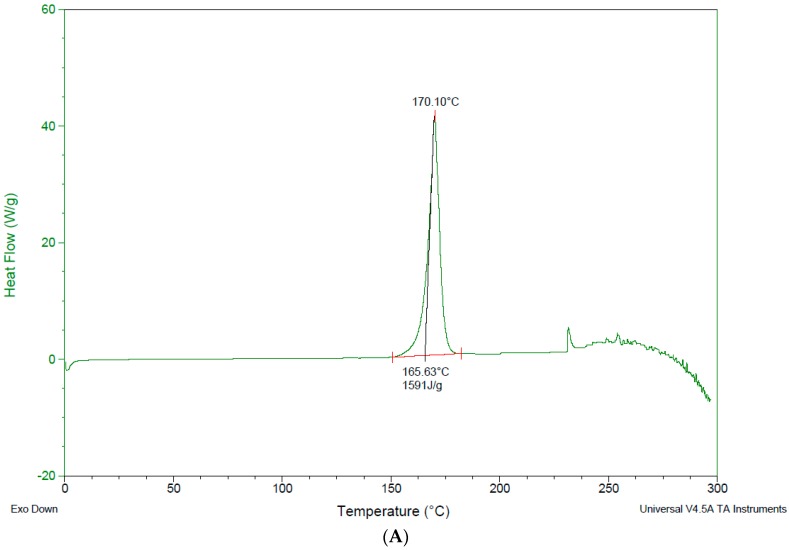

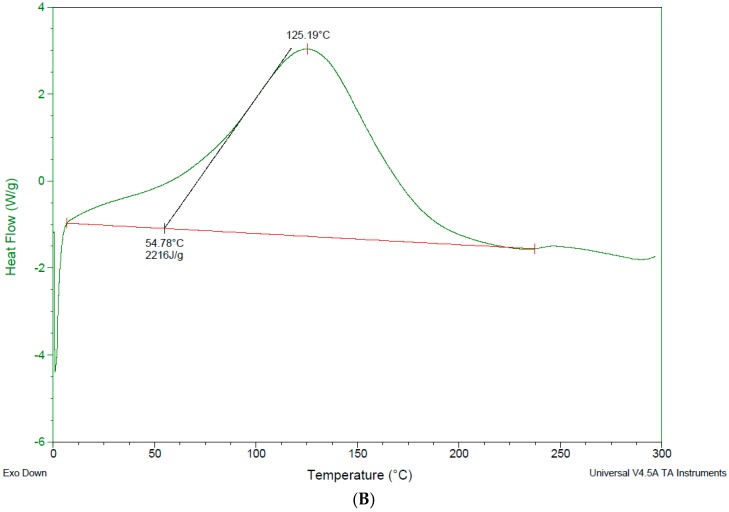

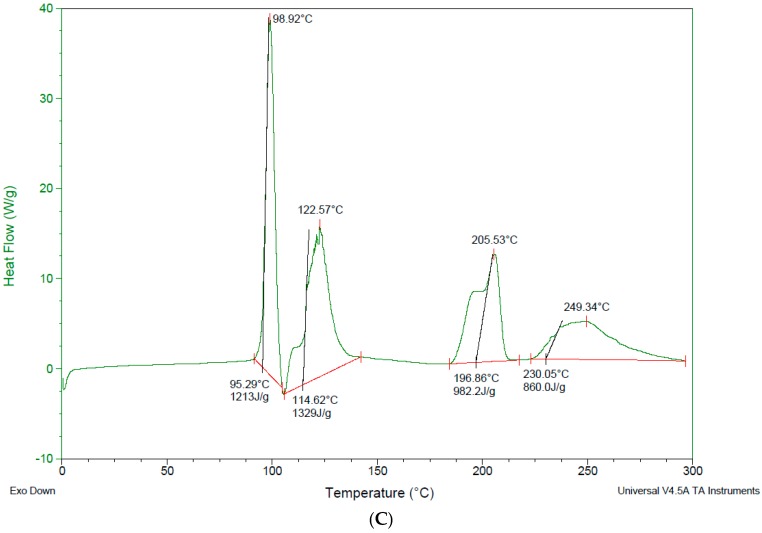
DSC data for pure excipients. (**A**) DSC for pure gluconolactone; (**B**) DSC for hydroxyl propyl γ-cyclodextrin; (**C**) DSC for trehalose.

For lyophilised samples (Figure 8), the peaks behaviours were different. In addition to *T*m and enthalpy reduction, some peaks disappeared and others were shown to melt with decomposition. The reason for this might be that the drug is completely solubilised in the formulations and molecularly dispersed within their excipients. This led to an improvement in the drug dissolution characteristics after addition of the excipients in different ratios. In drug-gluconolactone 1:1, 1:5, the drug peaks disappeared, for ratio 1:2 *T*m shifted down to 206.09 °C, and enthalpy to 188.1 J/g (Figure 8A) compared to the pure drug. For drug-cyclodextrin *T*m was reduced to 224.55 °C, 221.59 °C, 219.96 °C, and enthalpy was reduced up to 152.9 J/g, 87.78 J/g, 38,01 J/g for the ratio of 1:1, 1:2, 1:5 respectively (Figure 8B). DSC data for drug-trehalose samples (which are in agreement with the drug dissolution results versus pure drug) are shown in Figure 8C. DSC thermograms of the used excipients are shown in Figure 9 for comparison with those after inclusion of the drug in either physically mixed or lyophilized samples.

### 4.4. Scanning Electron Microscopy—SEM

**Figure 10:**
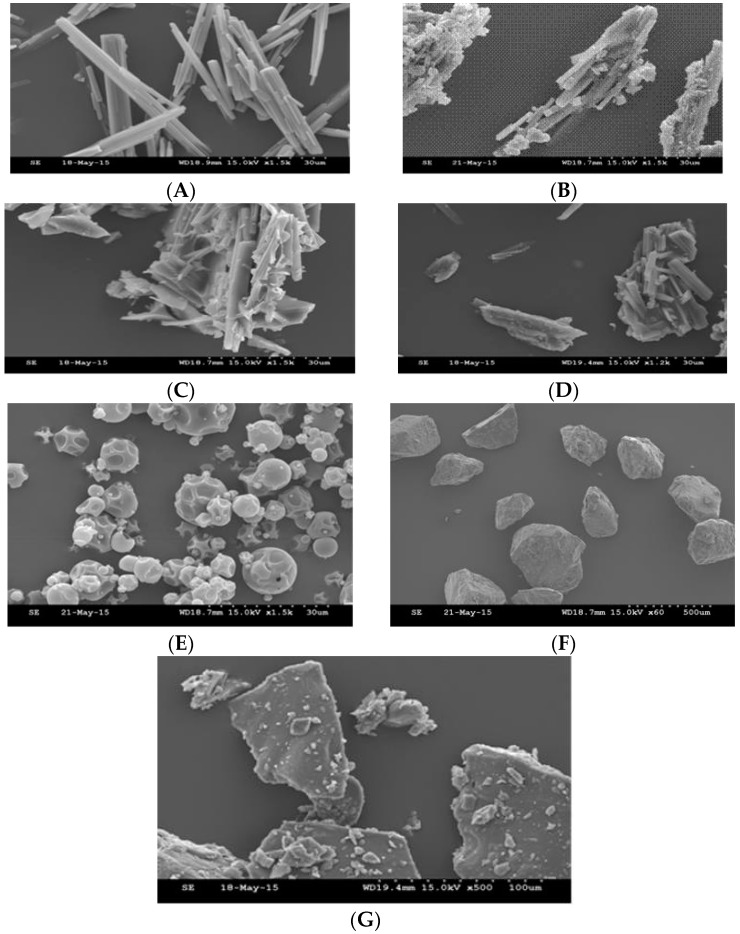
(**A**–**G**): Scanning Electron Microscopy (SEM) for lyophilized samples and pure excipients. (**A**) Pure drug; (**B**) Drug and gluconolactone 1:5; (**C**) Drug and hydroxyl propyl γ-cyclodextrin 1:5; (**D**) Drug and trehalose 1:5; (**E**) Pure hydroxyl propyl γ-cyclodextrin; (**F**) Pure gluconolactone; (**G**) Pure trehalose.

SEM (Figure 10) was used to investigate the morphology changes on the drug and used excipients, prior to and after lyophilisation to see the morphological changes after this process. The drug is a white powder, containing rectangular prism crystals [38]. Two excipients (gluconolactone and trehalose) are amorphous powders. The morphology of the drug and excipients does not show a big change in the shape of drug prism crystals in the amorphous state of the excipients (Figure 10A–G). This is a good indication that the drug was stable during the lyophilisation process, and did not greatly change, as it kept its crystallinity to a large extent after this process.

## 5. Conclusions

The used methods showed a significant enhancement in dug dissolution rate, *p* < 0.05. The dissolution studies showed that physical mixing significantly increased the percentage of drug release with time. Accordingly, it is feasible to apply physical mixing of this drug with the used excipients in the pharmaceutical industry and, hence, this will be cost effective. However, lyophilisation also enhanced the drug dissolution rate and the highest drug dissolution was for (drug-gluconolactone 1:1) with 98.98% release within 90 min, followed by (drug-cyclodextrin 1:5) with 97.23%, and the lowest drug dissolution extent was exhibited with (drug-trehalose 1:5) 93.18% drug release. Freeze drying is a lengthy process compared to the physical mixing. The used excipients and methods (physical mixing and freeze drying) proved their success at enhancing the dissolution rate of bendroflumethiazide and they might be promising methods and excipients that can be used to enhance dissolution of other hydrophobic drugs.
